# Evidence for the use of anti-CD20 therapeutic antibodies in immune thrombotic thrombocytopenic purpura management: a review

**DOI:** 10.3389/fphar.2026.1766030

**Published:** 2026-08-24

**Authors:** Konstantine Halkidis, Chan Meng, Jordan Snyder

**Affiliations:** 1 Department of Internal Medicine, Division of Hematologic Malignancies and Cellular Therapeutics, The University of Kansas Medical Center, Kansas, KS, United States; 2 Institute for Developmental and Reproductive Sciences, The University of Kansas Medical Center, Kansas, KS, United States

**Keywords:** antibody therapy, autoimmune, immune thrombotic thrombocytopenic purpura, thrombosis, treatment

## Abstract

Immune thrombotic thrombocytopenic purpura (iTTP) is an autoimmune thrombotic microangiopathy caused by antibody-mediated deficiency of the plasma metalloprotease ADAMTS13. Therapeutic antibodies that target antibody-producing B-cells via the CD20 surface receptor protein to prevent disease exacerbation and relapse have become part of the standard of care of patients with iTTP. However, clinical trial data regarding the use of anti-CD20 agents is limited to mostly phase II studies; no universally agreed-upon standardized protocol for the use of CD20-targeting antibodies for iTTP regarding choice of agent, dosage, or duration of treatment exists; refractoriness or lack of response to anti-CD20 antibodies can occur; and several agents and biosimilars are now available for the treatment of iTTP patients, which complicates the decision tree for clinicians caring for these patients. In this review, we explore the current landscape of anti-CD20 therapeutics used to treat iTTP, focusing on agents currently available for clinical use. We describe the pharmacokinetic and pharmacodynamic properties of CD20-targeting antibodies; available data regarding dosage and duration of treatment; anti-CD20 antibody therapy refractoriness or lack of response; and the evolving role of anti-CD20 biosimilars. This review will shed light on the state-of-the-art in CD20-targeting antibodies used to treat iTTP patients with an eye toward identifying areas in need of further study to aid in the care of patients with this rare and often devastating disease.

## 1 Introduction

**FIGURE 1:**
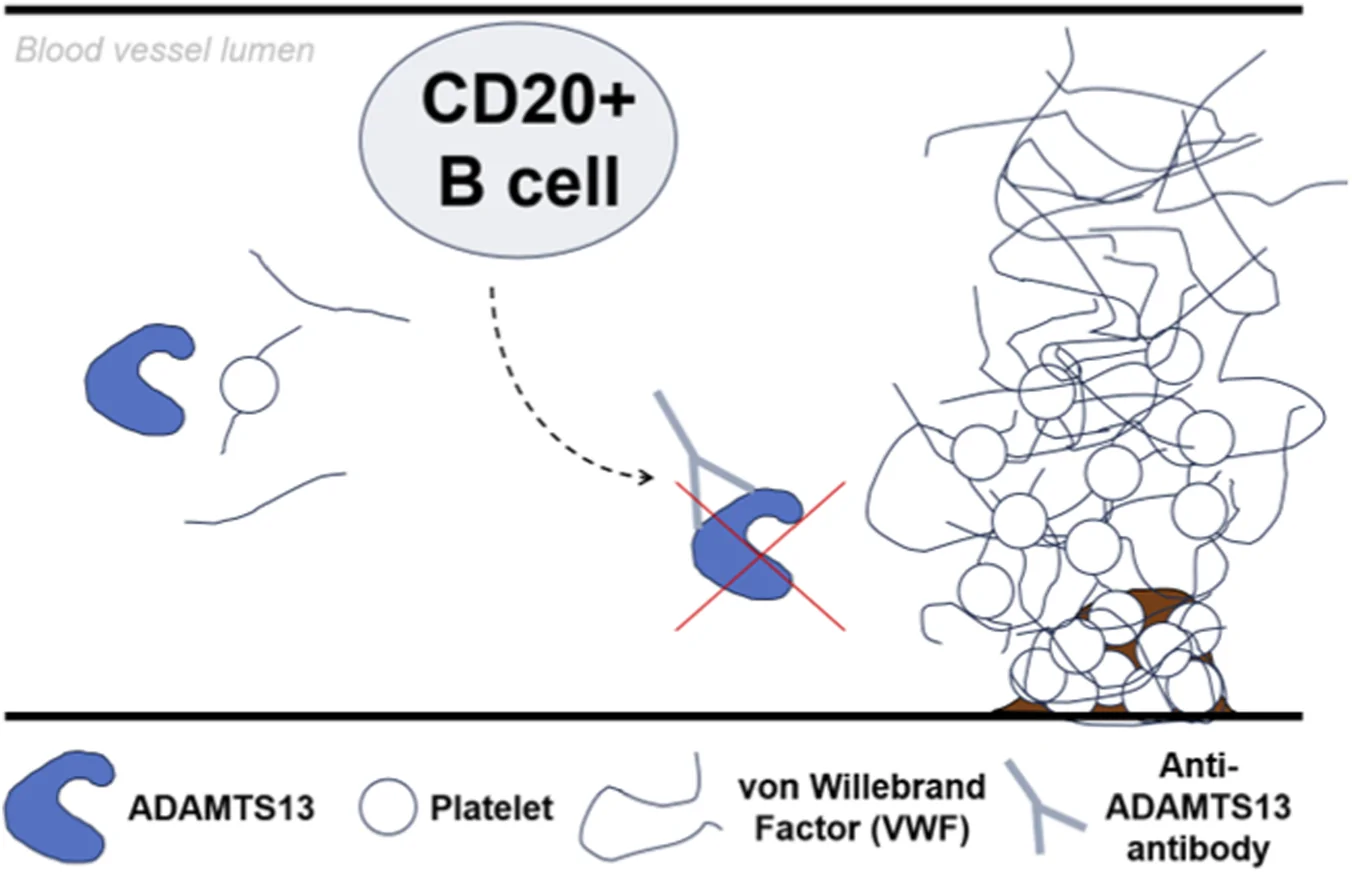
Simplified cartoon of the pathophysiology of immune thrombotic thrombocytopenic purpura. Plasma von Willebrand factor (VWF) (black linear objects), which bind to platelets (white circles) in the context of primary hemostasis to form an initial platelet plug. After initial platelet plug formation, ADAMTS13 (blue oblong object) cleaves ultra-large VWF multimers to relieve intravascular obstruction and restore laminar blood flow. In immune thrombotic thrombocytopenic purpura, antibodies (grey Y-shaped object) generated by CD20^+^ B-cell bind to ADAMTS13 and prevent VWF cleavage, bind to ADAMTS13 and prevent its function, leading to accumulation of ultra-large VWF multimers and vascular obstruction.

To this day, the antibody-mediated autoimmune disease iTTP is a hematologic emergency (Kessler et al., 2012). Mortality remains high unless there is a high index of suspicion and prompt initiation of treatment. The hallmarks of iTTP are thrombocytopenia; microangiopathic hemolytic anemia; deficiency of ADAMTS13, a plasma metalloprotease that contributes to primary hemostasis by cleaving and regulating the multimer size of von Willebrand factor (vWF), which binds to platelet GP1b receptors via the vWF A1 domain; and the presence of inhibitory anti-ADAMTS13 antibodies (Figure 1), the last of which is crucial to differentiate iTTP from the congenital form of TTP, since the treatment of each clinical entity differs significantly (Zheng et al., 2002). The two primary goals of treatment of patients with iTTP are: 1) restoration of activity of ADAMTS13, and 2) suppression of the production of anti-ADAMTS13 antibodies. The long-term goals of treatment are to achieve both clinical and ADAMTS13 iTTP remission, the former of which involves normalization of the platelet count and resolution of hemolysis, while the latter is generally defined as maintenance of the ADAMTS13 activity level above 20% (Zheng et al., 2020). The current standard of care is to begin therapeutic plasma exchange (TPE) as soon as the diagnosis is made or, if diagnostic ADAMTS13 assays are delayed, to begin empiric TPE while awaiting ADAMTS13 activity results if the probability of iTTP is sufficiently high in the estimation of the clinician (Zheng et al., 2020). Once diagnosis is confirmed in conjunction with detection of anti-ADAMTS13 inhibitory antibodies, immune modulation is pursued.

Immune suppression has been at the forefront of iTTP treatment modalities since the mid-20th century, when it was hypothesized that the reason patients only had a temporary improvement in their platelet counts when given plasma infusions was because a persistent inhibitor was present in patients’ blood (Shapiro et al., 1957). Initially, tools were limited in this regard, with corticosteroids being the primary modality utilized. However, in the modern era, clinicians have an array of options to choose from in terms of both restoring ADAMTS13 activity and keeping the pathogenic anti-ADAMTS13 antibodies at bay. In addition to corticosteroids, which are still widely used in the treatment of iTTP to this day, immunosuppressive treatments range from chemotherapeutic options such as calcineurin inhibitors [i.e., cyclosporine (Cataland et al., 2007)], purine synthesis inhibitors [i.e., mycophenolate mofetil (Fioredda et al., 2019), azathioprine (Bavli et al., 2021)], proteasome inhibitors [i.e., bortezomib (Shortt et al., 2013)], and alkylating agents [i.e., cyclophosphamide (Beloncle et al., 2012)]; surgical intervention [i.e., splenectomy (Beloncle et al., 2012)]; and therapeutic antibodies, upon which we expand below.

The ultimate goal of immunosuppression in iTTP is prevention of the production of anti-ADAMTS13 antibodies and their subsequent dissemination in the plasma while preserving as much beneficial immunity as possible. As such, the utility of treatments that preferentially affect antibody-producing cells of the immune system, such as B cells and plasma cells, is clear. The advent of B-cell targeting therapeutic anti-CD20 antibodies began with the first FDA approval of rituximab to treat relapsed or refractory low-grade non-Hodgkin’s lymphoma in 1997 (Anderson et al., 1997). Since then, treatment regimens for virtually all hematologic malignancies involving neoplastic CD20-expressing B cells include an anti-CD20 therapeutic antibody (Sehn and Salles, 2021; Willard et al., 2021). Many non-malignant antibody-driven autoimmune diseases are also treated with CD20-directed therapy (Kaegi et al., 2019). In the past decade, CD20-targeting antibodies have moved to the front line of iTTP treatment, as they significantly reduce the risk of iTTP exacerbation and relapse (Chaturvedi et al., 2022; Kubo et al., 2020; Owattanapanich et al., 2019; Sun et al., 2019).

With the new and evolving landscape of therapeutic antibodies, clinicians caring for iTTP patients must weigh the risks and benefits of each treatment in terms of efficacy, adverse effects, the influence of comorbid conditions, and the relative availability of these agents. However, limited clinical trial data is available to guide management decisions regarding optimal choice of CD20-targeting agent, dosage, or duration of treatment. Though generally well tolerated, anti-CD20 therapeutic antibodies have been associated with life-threatening drug-related adverse events and can be logistically challenging to administer (Fenton et al., 2020). The landscape for physicians and researchers seeking to utilize widely available, efficacious iTTP treatment approaches including CD20-targeting therapeutics that minimize toxicity remains complex. In this article, we will review the available state-of-the-art evidence regarding the utilization of anti-CD20 antibodies in the care of iTTP patients. We describe the pharmacodynamics and pharmacokinetics of CD20-targeting antibodies, and how they differ amongst the available agents; explore the data regarding efficacy and adverse events of agents and regimens used in the treatment of iTTP with anti-CD20 antibodies from clinical trials and other literature reports; and consider the emerging roles of CD20-targeting biosimilar antibodies in autoimmune diseases such as iTTP. We discuss the future of CD20-targeting agents in iTTP and the need for further studies to help develop standardized approaches to their utilization.

## 2 Methods

Literature was searched using PubMed from 1997 to 2025 using free text searches including “thrombotic thrombocytopenic purpura” associated with the Boolean operater “AND” in combination with the following free text phrases: “rituximab”, “CD20”, “obinutuzumab”, “ofatumumab”, “ocrelizumab”, “ublituximab”, “rituximab-abbs”, “rituximab-pvvr”, “rituximab-arrx”, “ruxience”, “riabni”. We also utilized the following free text phrases to explore information on anti-CD20 therapy outside of the context of iTTP (“rituximab”) AND (“subcutaneous”) ((“Obinutuzimab”) OR (“ofatumumab)) AND (“autoimmune”) (“rituximab”) AND (“biosimilar”). We found 1,737 peer-reviewed articles and sorted through these for literature pertinent and relevant for the review. A total of 141 articles were included for review and the remainder were excluded.

## 3 Biochemical and biophysical properties of anti-CD20 antibodies

### 3.1 General mechanistic effects of CD20-targeting antibodies

**FIGURE 2:**
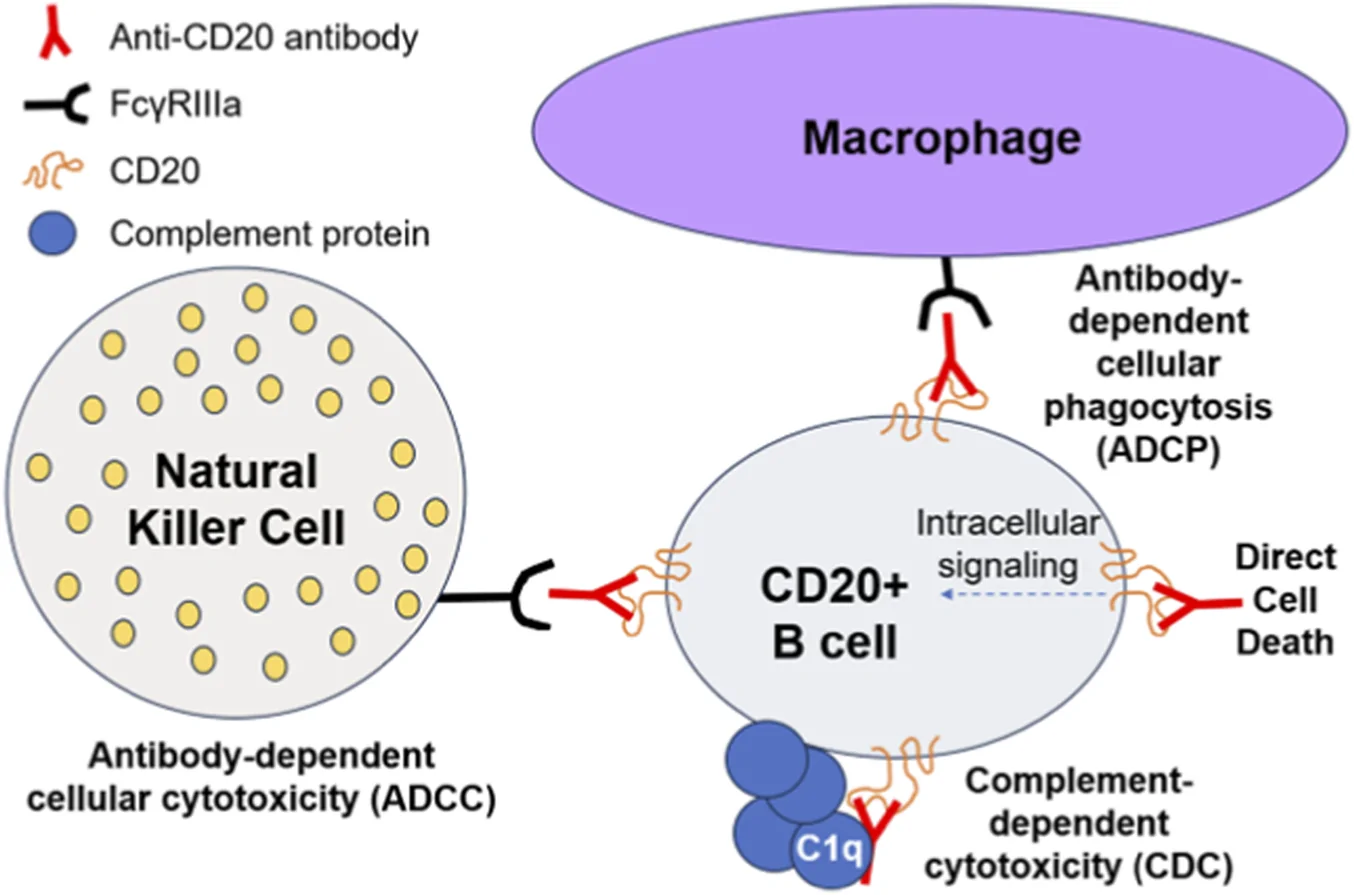
Mechanisms of action of anti-CD20 therapeutic antibodies. Binding of anti-CD20 antibodies (red Y-shaped object) to the CD20 receptor on B cells (orange linear objects) can induce cell death via various mechanisms, including direct cell death via intracellular signaling after CD20 binding; binding of the Fc region of the anti-CD20 antibody to FcγIIIa receptors (black objects) on macrophages to induce antibody-dependent cellular phagocytosis (ADCP), or to natural killer cells, which can induce antibody-dependent cellular cytotoxicity (ADCC); and complement-dependent cytotoxicity (CDC) via interaction of the anti-CD20 CH2 region of the Fc portion of the antibody with complement proteins (blue circles), primarily C1q, and subsequent activation of the complement system to induce cell death.

CD20 is expressed by mature B-lymphocytes but not by hematopoietic stem cells, pro-B-cells, or terminally differentiated plasma cells, and CD20-targeting antibodies can suppress B cells by a variety of mechanisms (Figure 2) (Athni and Barmettler, 2023). Once bound to CD20^+^ cells, they can trigger complement dependent cytotoxicity (CDC) and induction of natural killer (NK) cell-mediated antibody-dependent cellular cytotoxicity (ADCC) via Fc interaction with the FcγRIIIa of NK cells. In addition to these “immune-mobilizing” mechanisms, anti-CD20 antibodies can directly induce cell death via intracellular signaling; inhibit cell proliferation; induce antibody-dependent cellular phagocytosis (ADCP), in which macrophages recognize antibody-coated target cells (also via FcγRIIIa) and subsequently phagocytose the antibody-bound B cells; and sensitize cells to chemotherapeutic agents. Though these general principles apply to CD20-targeting inhibition of B-cell, predominant mechanistic effects differ amongst the various anti-CD20 antibodies available for clinical use.

### 3.2 Mechanistic differences amongst anti-CD20 antibodies

**FIGURE 3:**
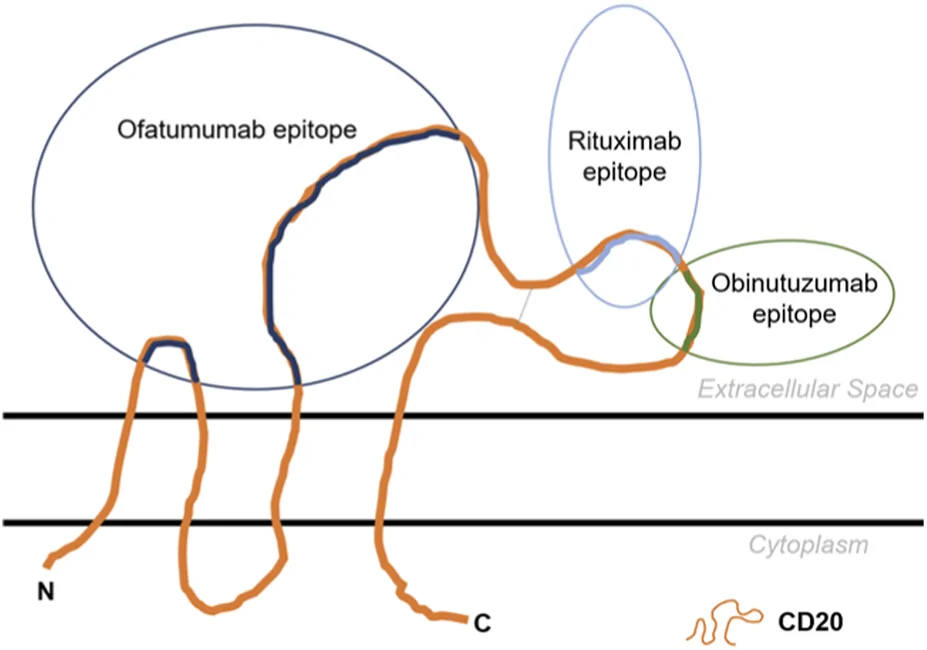
Binding epitopes of anti-CD20 therapeutic antibodies. The transmembrane extracellular CD20 receptor (orange linear object) on B cells is bound by anti-CD20 antibodies at different epitopes. The ofatumumab binding epitope (dark blue) contains amino acid residues in both the small and large extracellular loop of CD20, while rituximab (light blue) and Obinutuzumab (green) bind to the large extracellular loop.

Several CD20-targeting therapeutic antibodies have been developed for the treatment of B-cell neoplasms and autoimmune diseases. Rituximab is a partially humanized chimeric monoclonal anti-CD20 antibody, the first in its class to be developed and approved for clinical use that binds to the large extracellular loop of CD20 (Figure 3) (Teeling et al., 2006). Though traditionally administered intravenously, a subcutaneous rituximab formulation was approved by the FDA in 2017 (Yelvington, 2018; Aue et al., 2010; Mao et al., 2013), and more recently biosimilar antibodies with the same amino acid sequence as rituximab have become available (Vital et al., 2013). Obinutuzumab, fully humanized, binds to an epitope that partially overlaps with that of rituximab on the large extracellular CD20 loop (Lee et al., 2014; Niederfellner et al., 2011). Ofatumumab is a fully humanized monoclonal antibody with a binding epitope distinct from that of either rituximab or Obinutuzumab involving the small and large loops of CD20 (Lin, 2010).

Anti-CD20 antibodies can be characterized as type I (rituximab, ofatumumab) or type II (Obinutuzumab) immune effectors (Table 1). For the type I effectors, CDC via binding of C1q to the CH2 region of the monoclonal antibody Fc region is higher than for Obinutuzumab (Vidarsson et al., 2014; Kovacs et al., 1998; Klein et al., 2013). Each of these antibodies is capable of inducing ADCC and ADCP, but as a type II antibody with an Fc region engineered to have increased affinity for FcγRIIIa, ADCC and ADCP are higher for Obinutuzumab, and it also induces cell death more potently than type I antibodies (Niederfellner et al., 2011; Robak, 2009; Patz et al., 2011). Though both rituximab and ofatumumab are type I antibodies, the binding affinity of Ofatumumab for CD20 is higher than that of rituximab, making it less dependent on cell surface CD20 receptor density; additionally, ofatumumab-induced complement activation is higher compared to rituximab (Lin, 2010; Teeling et al., 2004; Uchiyama et al., 2010). Since Obinutuzumab and ofatumumab are humanized and fully human, respectively, rates of infusion reaction and anaphylaxis are lower than for rituximab, the latter of which can lead to development of human anti-chimeric antibodies and infusion reactions in 10%–15% of patients (Masoud et al., 2018; Lizondo et al., 2025; Crowley et al., 2018; Teisseyre et al., 2025). We will expand on these differences in drug-related adverse events in a clinical context in subsequent sections of this review.

**TABLE 1:** Characteristics of anti-CD20 antibodies.

Origin	Rituximab	Obinutuzumab	Ofatumumab
	Chimeric	Humanized	Human
Type	I	II	I
ADCC	↑	↑↑↑	↑
ACDP	↑	↑↑↑	↑
CDC	↑	-	↑↑↑
Direct cell death	↑	↑↑↑	↑

Relative differences in the mechanistic characteristics of rituximab, Obinutuzumab, and ofatumumab. ADCC, antigen-dependent cellular cytotoxicity; ADCP, antigen-dependent cellular phagocytosis; CDC, complement dependent cytotoxicity.

Rituximab represents the drug in the class with alternative formulations used to treat patients with iTTP. Subcutaneous formulations of rituximab appear to have similar efficacy in non-Hodgkin’s lymphoma with pharmacokinetics comparable to that of IV rituximab, though less data is available for its utilization to treat non-neoplastic conditions (Yelvington, 2018; Delrue et al., 2021; Bittner et al., 2014; Davies et al., 2014). In recent years, biosimilar biologics, defined as “highly similar to the reference product notwithstanding minor differences in clinically inactive components” for which no “clinically meaningful differences between the biological product and the reference product in terms of safety, purity and potency,” have come into clinical use around the world (Kay, 2011; Kozlowski et al., 2011). Biosimilar therapeutic antibodies have the same primary amino acid sequence of the original but are reverse-engineered based on the composition of the original antibody and tested using various techniques to demonstrate their similarity to the original drug. These drugs are essentially mechanistically and pharmacokinetically indistinguishable from the original formulation and their biophysical profiles closely match as well, though due to the technical complexity of biotherapeutic manufacturing, they are never exactly identical to the original product (Kozlowski et al., 2011). Several rituximab biosimilars are available for clinical use (Shelbaya et al., 2021), including three available in the United States: rituximab-abbs (Wong et al., 2024; Barke et al., 2023; McBride A. et al., 2021), rituximab-pvvr (Ryan et al., 2014; Cohen et al., 2016), and rituximab-arrx (Cobb et al., 2022; McBride H. J. et al., 2021; Burmester et al., 2020; Niederwieser et al., 2020). However, literature regarding the use of these biosimilars for the treatment of iTTP is lacking and the adverse event profiles of biosimilars of rituximab do appear to differ from the original IV formulation, as we relate in greater detail later in this work.

## 4 Clinical utilization of CD20-targeting antibodies to treat iTTP

### 4.1 Rituximab

When iTTP was recognized as a polyclonal antibody-mediated autoimmune disease, researchers and clinicians recognized the potential benefit of CD20-directed therapy for the disease; initially, the agent was reserved for the treatment of patients with relapsed or refractory iTTP. Rituximab was first reported as a potential therapeutic option for iTTP in 2002 b y a group in Germany (Chemnitz et al., 2002). The authors described two female patients with refractory iTTP despite multiple lines of therapy who responded well to rituximab at a dose of 375 mg/m^2^ while continuing TPE, with one patient receiving four weekly doses and the other two weekly doses. Multiple case reports were subsequently published in the following few years (Gutterman et al., 2002; Yomtovian et al., 2004; Zheng et al., 2003; Tsai and Shulman, 2003; Fakhouri et al., 2004), eventually leading to clinical trials to evaluate the effectiveness and tolerability of rituximab for the treatment of iTTP in a controlled manner. By 2012, accumulating evidence suggested that rituximab significantly improved rates of remission from iTTP in patients with “acute refractory or chronic relapsing idiopathic” iTTP (Tun and Villani, 2012), and in the following years, rituximab moved into the front-line preemptive setting (Jestin et al., 2018). When rituximab is given intravenously during acute iTTP episodes, the drug is ideally administered immediately after TPE to maximize exposure prior to the next exchange (Zwicker et al., 2019; Fauvelle et al., 2000; Darabi and Berg, 2006). Below, we review the available evidence regarding efficacy, adverse effects, dosage, formulation, refractoriness, and resistance to rituximab therapy in iTTP.

#### 4.1.1 Clinical trials exploring the use of rituximab in iTTP

In this section, we review the results of clinical trials to determine the utility of rituximab in treating iTTP (Table 2). The first multicentric open-label prospective trial to test the efficacy of rituximab in both the acute setting and as prophylaxis was conducted at five institutions in France and Italy in 2005 (Fakhouri et al., 2005). Eleven patients with either acute iTTP (n = 6) or in clinical remission from the disease (n = 5) were treated with rituximab at a dose of 375 mg/m^2^ for 4 weeks. Patients with acute disease were concomitantly treated with TPE and steroids. All patients had previously been treated with at least one iTTP-directed immunosuppressive agent, with eight of the patients previously receiving vincristine and six patients with prior splenectomy. Notably, two of the patients had previously received rituximab, though each of these two patients was in clinical remission at the time of enrollment. For patients receiving rituximab in the acute setting, all achieved clinical remission 5–14 days after the fourth rituximab infusion, and all patients had recovery of ADAMTS13 activity to levels >10% by 6-month post-rituximab, though notably three patients with acute iTTP and one patient in clinical remission at the time of enrollment had ADAMTS13 activity levels <5% at the 3-month mark. None of the patients experienced iTTP relapse during the study. One patient experienced mild hypotension with the first rituximab treatment which resolved after infusion rate was decreased. Overall, the data was encouraging and supportive of the hypothesis that rituximab is a well-tolerated and effective treatment for iTTP in both the acute setting and as a prophylactic agent.

**TABLE 2:** Clinical trials to explore the utility of rituximab in the treatment of iTTP.

Study References	Year of publication	Patient population	Intervention	Selected outcomes
Fakhouri F, et al. Pmid 15,933,059	2005	Current acute refractory TTP episode (n = 6)	Rituximab 375 mg/m^2^ weekly x4 doses + TPE + corticosteroids	Rituximab induced a clinical remission in 5–14 days after 4th infusion
Scully M, et al. Pmid 21,636,861	2011	Current acute TTP (n = 40)	Rituximab 375 mg/m^2^ weekly x4 doses + TPE + corticosteroids	Median number of TPE treatments: 16.5 (range: 4–34) vs. 18 (range: 6–92) in historical control (p = 0.5)
Froissart A, et al. Pmid 21,926,591	2012	Refractory acute TTP episode (n = 22)	Rituximab 375 mg/m^2^ x4 doses given over 15 days + TPE	Time to durable remission was significantly shorter versus historical controls (p = 0.03)
Clark W, et al. Pmid 25,855,259	2015	Relapsed and/or refractory TTP (n = 40)	Rituximab 375 mg/m^2^ weekly x4 doses + TPE + corticosteroids	Complete response was achieved in 70% of patients with refractory disease and 80% of patients with relapsed disease by week 8
Miyakawa Y, et al. Pmid 27,188,338	2016	Relapsed/refractory TTP (n = 6)	Rituximab 375 mg/m^2^ weekly x4 + TPE	33.3% (2/6) of patients achieved a platelet count >150 × 10^9^/L at week 4
Benhamou Y, et al. Pmid 27,643,485	2016	Acquired TTP with a suboptimal response to TPE (n = 24)	Rituximab 375 mg/m^2^ up to 3 doses on days 1, 4, and ± 15	Time to platelet recovery was similar with 3 doses of rituximab versus historical control with 4 rituximab doses (p = 0.82)
Zwicker J, et al. Pmid 3,1,331,919	2019	Acute TTP (n = 19)	Rituximab 100 mg administered every 5–9 days x4 weeks + TPE + corticosteroids	A cumulative incidence of exacerbation or refractory TTP at 30 days was 12.5%

Summary results from clinical trials of rituximab treatment for iTTP, patients. iTTP: immune thrombotic thrombocytopenic purpura; TPE: therapeutic plasma exchange.

In 2011, results from a nonrandomized phase two study to assess the safety and efficacy of rituximab in conjunction with TPE were published by a group from the United Kingdom, specifically the South East England TTP study group (Scully et al., 2011). All patients had acute iTTP at the time of enrollment, either *de novo* or relapsed disease, and were treated with rituximab 375 mg/m^2^ IV with four weekly doses, with the first dose given within the first 3 days of admission and diagnosis. A total of 40 patients were enrolled and followed for at least 12 months. Compared to historical controls, rituximab helped reduce the duration of hospitalization by 7 days in non-ICU patients; reduced relapse rate (10% vs. 57%); and patients receiving rituximab did not experience serious adverse events.

Also during the year 2011, the French Thrombotic Microangiopathies Reference Center published the results of an open-label prospective study exploring the efficacy and safety of first-line rituximab in patients with “severe” iTTP with a suboptimal response to TPE (Froissart et al., 2012). Twenty-two adults with no response or iTTP exacerbation when treated with TPE received four infusions of rituximab 375 mg/m^2^ IV over 15 days, initiated on “the day a suboptimal response was diagnosed” and given on days 0, 3, 7, and 14 due to concern that ongoing TPE may have affected the circulating concentration of rituximab in patient plasma. One patient died within a week of rituximab initiation in the context of continued iTTP exacerbation, but all 21 survivors experienced platelet count recovery within 35 days compared to 78% from a cohort of historical controls. Rituximab was effective in suppressing circulating CD19 (+) B cells for 9 months, though this effect was lost by the 12 months mark. Similarly, B cell depletion induced by rituximab correlated with higher ADAMTS13 activity and lower ADAMTS13 antibody titers. No adverse events related to rituximab were reported.

In 2015, members of the Canadian Apheresis Group reported their findings from a phase two sequential case-series study to determine the efficacy of rituximab in the setting of treating adult patients with relapsed or refractory iTTP (Clark et al., 2015). A total of 40 patients, 20 apiece with either refractory or relapsed iTTP, were enrolled and were administered rituximab 375 mg/m^2^ IV in four weekly doses in conjunction with TPE and steroid therapy standard at the time. In the intention-to-treat analysis, 75% of patients with refractory disease were alive and in clinical remission at 52 weeks, with 70% achieving a complete response by week 8; 90% of patients with relapsed disease were alive and in clinical remission at 52 weeks, with 80% having achieved remission by week 8. However, only 38 of the 40 patients had ADAMTS13 testing done at the time of study entry. 31.6% and 47.3% of patients with refractory and relapsed disease, respectively, had initial ADAMTS13 activity levels >10%, suggesting that at least some of the patients in the cohort, and perhaps a significant number, may not have had iTTP. Notably, two patients died of sepsis due to infection during the study, thought by the authors to be potentially at least partially attributable to immunosuppression from steroids and rituximab.

In 2016, a Japanese open-label, single-arm multicenter study was published which explored the efficacy and safety of rituximab in iTTP patients with relapsed or refractory disease (Miyakawa et al., 2016). Rituximab was administered at a dose of 375 mg/m^2^ IV in four weekly doses in conjunction with standard treatment including TPE and steroids. Though a relatively small study which included only six patients deemed “evaluable” in the final analysis, the authors reported clinically significant recovery of platelet count with discontinuation of TPE, ADAMTS13 activity recovery, ADAMTS13 inhibitor suppression to levels below the lower limit of detection, and improvement of clinical manifestations of iTTP in all patients. No safety issues were identified with administration of rituximab.

In the same year of 2016, a French group reported results from a single-arm, prospective, multicenter phase II study intended to help refine the dosing of rituximab necessary to deplete circulating B cells in patients with iTTP (Benhamou et al., 2016). With a primary endpoint of time to platelet count recovery from first plasma exchange, patients were administered rituximab 375 mg/m^2^ IV, initiated on “the day a suboptimal response was diagnosed” and with repeat administration on day +4. On day +14, flow cytometry to detect the number of circulating CD19 (+) B cells in patients was performed, with a decision on whether to administer a dose rituximab on day +15 based on whether such B cells were detectable at that time. All patients received an additional dose of rituximab on day +30. Results were compared to historical results from patients from the 2011 study who all received four total doses of rituximab as described above. Overall, 24 patients were enrolled, with 21 having *de novo* disease and 3 with “a past history” of iTTP; 14 patients received two initial doses, and 10 patients received three initial doses based on CD19 (+) B cell flow cytometry results day +14. Though direct comparison could not be done, meaning that the timing of the flow cytometry did not precisely match between the study group and the historical group, all surviving patients did have undetectable circulating B cells at 1 month and 3 months after study initiation. Three patients died during the study, all within approximately a week of rituximab initiation and all attributed to complications of iTTP rather than adverse events related to intervention. No serious safety events were reported.

Considering the efficacy and durability of response observed in clinical trials, investigators began to speculate that alternative dosing regimens that do not expose patients to as high a dose of rituximab may be equally beneficial while mitigating adverse effects. In 2019, results of a prospective, multicenter, single-arm, phase II trial exploring the efficacy of adjuvant low dose rituximab plus plasma exchange in the treatment of iTTP patients compared to historical data from patients receiving standard dose rituximab were published, with a composite outcome of exacerbation or refractory iTTP as the primary endpoint (Zwicker et al., 2019). Patients were administered 100 mg/m^2^ rituximab IV “every 5–9 days” over a period of 4 weeks. Treatment was given concurrently with TPE as a prerequisite, and initiation of rituximab was required to begin before five total TPE sessions had been completed. A total of 19 patients were initially enrolled but only 18 received rituximab because one patient was found to have congenital TTP and not iTTP. Results were similar to previous findings using the 375 mg/m^2^ dose, with only one patient experiencing a relapse within a year of treatment initiation, two additional relapses between years one and 2, and a relatively benign safety profile. The investigators did report one patient experienced a grade 4 non-fatal episode of respiratory failure during infusion of the third dose of rituximab and another patient died in the setting of advanced cancer 18 months after enrollment, though this was thought to be unrelated to rituximab.

#### 4.1.2 Alternative rituximab dosing regimens for iTTP

Clinicians caring for iTTP patients in the modern era have several different rituximab dosing regimens to choose from. In this section, we present available data on alternative dosing regimens in addition to the result from the 2019 trial described above. A case series of four patients who received a flat dose of 100 mg IV rituximab weekly for 4 weeks was reported in 2013; three patients were treated with up-front rituximab, while one patient received rituximab as salvage therapy (Pequeno-Luevano et al., 2013). All four patients experienced a lasting remission with no relapses between the iTTP episode treated with this rituximab dosage and a range of 8–22 months by the time of publication. The 100 mg flat dose was also reportedly successful in achieving lasting remission in multiple other publications, including a 2015 report of two patients who received rituximab as salvage therapy (Tong et al., 2015). A 2016 analysis of 10 patients with a first iTTP acute episode and one patient with relapsed disease treated with the 100 mg rituximab dose also showed good efficacy, with an estimated 2-year relapse-free survival reported to be 89% (Vazquez-Mellado et al., 2016). A 2020 report of two patients with iTTP associated with systemic lupus erythematosus (SLE) and treated with 100 mg rituximab IV weekly for 4 weeks for refractory iTTP each experienced a lasting remission from both iTTP and SLE flares during 24 months of follow up (Ma et al., 2020). One report from a group in China considered a different strategy of administration of a single dose of rituximab 375 mg/m^2^ IV to be considered as an alternative up-front regimen in developing nations in which weekly administration for 4 weeks may not be logistically feasible; of five patients treated with the 375 mg/m^2^ single dose with initially diagnosed iTTP and two patients with relapsed disease, relapse-free survival at 2 years was 83.3% (Tan et al., 2022).

However, one study demonstrated that, at least for some patients, the standard dose of 375 mg/m^2^ IV weekly for 4 weeks may be of greater benefit (Reddy et al., 2020). A retrospective analysis in 2020 b y a group from the University of Texas Southwestern Medical Center of 39 patients treated for iTTP over a 13 year period compared outcomes of patients who received standard 375 mg/m^2^ rituximab IV for 4 weeks (17 patients) to those who received the fixed 100 mg alternative regimen (8 patients). There was some variability between the standard dose and the low dose groups, with inhibitor titer being slightly but significantly higher in the standard dose group, and the patients receiving standard rituximab for preemptive treatment while in clinical iTTP remission having a lower median pre-course ADAMTS13 activity. The patients receiving the low-dose regimen had a shorter time to relapse compared to the standard dose group (7.5 months vs. 10 months) which was statistically significant. Only one patient in the standard dose group experienced a clinical relapse within active follow up, compared to three patients in the low-dose group. Notably, several of the patients did not receive four total treatments per rituximab treatment course (7 patients in the standard dose group, three patients in the low dose group). For the patients who were specifically administered rituximab for preemptive treatment during iTTP remission (7 patients in the standard dose group, six patients in the low dose group), outcomes were essentially the same, with only a statistically significantly higher median ADAMTS13 activity level at 1 month in the low dose group. The reader should caution, however, that this is a retrospective study and several variables may have contributed to the heterogeneity of outcomes as has hopefully been made clear from our description here.

Overall, several different rituximab dosing regimens appear to be reasonable, safe, and efficacious for the treatment of iTTP patients. Studies to date have mostly been limited by small numbers of patients, confounding variables between groups, and the retrospective nature of most of the available data. However, as iTTP is an autoimmune disease and not a neoplasm, it is reasonable to treat patients with regimens more akin to those used for patients with autoimmune disorders. A common dosing regimen is 1,000 mg rituximab given intravenously on days 1 and 15 in diseases like rheumatoid arthritis (Bertrand et al., 2025), autoimmune hepatitis (Burak et al., 2013) and Henoch-Schonlein purpura (Pindi Sala et al., 2014), as well as hematologic autoimmune disorders like autoimmune hemolytic anemia (Liu and Cheuk, 2021) and immune-mediated thrombocytopenia (Khellaf et al., 2014; Tran et al., 2014; Mahevas et al., 2013).

#### 4.1.3 Other evidence for the use of standard and alternatively dosed rituximab in iTTP to preempt disease recurrence and engender lasting remissions

Higher quality phase II clinical trial evidence regarding the use of rituximab is essentially limited to the studies described in a previous section. However, due to the favorable toxicity profile of rituximab and evidence of its efficacy first being published in the context of a trial in 2005, several groups around the world began to incorporate rituximab into their iTTP treatment approach since the mid-2000s (Stein et al., 2011; Ojeda-Uribe et al., 2010; Chemnitz et al., 2010). In 2014, the French Thrombotic Microangiopathies Reference Centre group published a cross-sectional analysis of 12 years of follow up data from 2000 to 2012 to compare relapse rates among patients who received rituximab in the setting of ADAMTS13 activity <10% during clinical remission to those who did not receive rituximab in this context (Hie et al., 2014). In total, 30 patients received rituximab, while 18 did not in this scenario. Relapse-free survival was significantly longer in the group that received rituximab compared to the group that did not, though notably eight of the patients in the latter group were considered to be diagnosed and treated for iTTP in the “pre-rituximab era” and the other 10 were treated at centers where preemptive rituximab was not considered standard of care. There was also heterogeneity in terms of the number of acute iTTP episodes experienced by each group. Whether other treatments were considered or utilized in the preemptive setting is not discussed in the manuscript or supplementary materials, though towards the latter point the authors do state that patients were all treated according to consensus French national guidelines in all other ways except for the use of preemptive rituximab.

In 2016, members of the Oklahoma TTP Registry reported their experience treating iTTP patients between 2003 and 2017 with rituximab (Page et al., 2016). Out of 41 total patients who experienced their first episode of iTTP in this time period, 37 survived the initial acute episode; one survivor was subsequently lost to follow up. Of the remaining 36 patients, 16 patients were treated with rituximab, most often (14/16) with 375 mg/m^2^ IV weekly for 4 weeks; the two other patients received only one infusion of 375 mg/m^2^. Notably, the patients who had received rituximab tended to require additional plasma exchange over a longer duration as well as a greater total amount of steroids compared to the non-rituximab treated patients. Nonetheless, despite this evidence that the rituximab-treated group likely had more severe disease, the investigators found that rituximab significantly improved relapse-free survival.

In 2017, iTTP patients identified from the United Kingdom TTP Study Registry that were administered rituximab for an episode of acute iTTP were analyzed retrospectively with respect to the dose of rituximab utilized and correlation with iTTP-directed treatment free survival (Westwood et al., 2017). Out of 76 patients identified from 2005 to 2016 in the registry, 24 received the “standard” dose of 375 mg/m2 IV weekly for 4 weeks; 19 received a “reduced” dose of 200 mg IV weekly for 4 weeks; 17 received an “intermediate” dose of 500 mg IV weekly for 4 weeks; and 16 patients received “other” doses ranging from “100–1,000 mg rituximab in one–five doses”. The study found that no dose conferred a statistically significant difference in treatment free survival, though “reduced” dose patients did have a shorter time to re-treatment with rituximab. Notably, this re-treatment was most often associated with prophylactic administration of rituximab given for an ADAMTS13 activity level <15% rather than clinical iTTP relapse, comprising 35/38 total cases of patients treated with another course of rituximab. Though reassuring regarding support for the utilization of lower doses of rituximab, this study and several others described here demonstrate the heterogeneity of the data in the literature, even within a given study.

A group from Germany and Switzerland reported their findings in 2018 regarding relapse rates amongst survivors of iTTP treated with or without rituximab (Falter et al., 2018). In a retrospective analysis of 70 patients diagnosed with iTTP between 2003 and 2014 at their institutions, they identified 45 of whom experienced their first episode in that time period; 17 patients received rituximab 375 mg/m^2^ IV weekly for 4 weeks, and 28 did not receive rituximab. Though there was a fairly clear trend toward improved relapse-free survival when patients were followed for a median of 8.3 years, this did not rise to the level of statistical significance. In that same year, the French Reference Center for Thrombotic Microangiopathies group published additional results on the preemptive use of rituximab in iTTP patients (Jestin et al., 2018). Here again, preemptive treatment was defined as administration of rituximab in the context of a low or declining ADAMTS13 activity level in patients who had previously experienced episodes of acute iTTP but who did not have clinical iTTP relapse at the time of rituximab administration. A total of 92 patients were administered preemptive rituximab in a cohort that included patients who were added to the group’s registry between 2012 and 2017, where preemptive rituximab became standard of care for the group, as well as patients previously administered preemptive rituximab as described in earlier reports. Relapse rates were compared to 23 historical controls, a group that included patients treated before the rituximab era and those treated at centers where preemptive rituximab was not routinely administered in this context. The rituximab group had remarkably and significantly lower relapse rates compared to the historical control group, with 15% and 74% of patients experiencing clinical relapse in the two groups “after a 7-year follow-up”, respectively. No treatment-altering events were reportedly associated with rituximab administration, though 20.7% of the cohort experienced “benign” adverse effects.

In 2019, a group analyzed iTTP relapse rates amongst a multi-institutional cohort of 124 consecutive adult iTTP patients between 2004 and 2017 stratified according to whether or not they were treated with rituximab for the index episode (Sun et al., 2019). The investigators defined “index” episodes as those occurring for the first time at their institution regardless of whether the patients had previous iTTP episodes at other institutions. Significantly fewer relapse episodes at 1 year of follow up occurred amongst patients who received rituximab, though the iTTP relapse rate at 5 years of follow up was not significantly different between the groups. The investigators noted that age <25 years at presentation, non-O blood group, and presentation in iTTP relapse of patients included in the study were predictors of subsequent relapsed iTTP episodes. Notably, the vast majority (97%) of the rituximab-treated patients were treated with a dose of 375 mg/m2 IV, but only 86% of these patients received four weekly doses; the precise number of doses for the other patients was not reported. Two patients were treated with 100 mg IV rituximab, but only one of the two received four weekly doses, and again, the precise number of doses for the other patient was not reported. Only the first relapse after the “index” episode was analyzed. Data regarding adverse events was not reported in this study.

The French consortium began offering subcutaneous (SQ) rituximab/hyaluronic acid administered as a single dose of 1,400 mg in the preemptive setting to patients with iTTP in 2015, and published their findings in 2020 (Delrue et al., 2021). Twelve patients elected for the SC formulation over that period of time, with diverse treatment plans in terms of total number of doses administered depending on persistence of ADAMTS13 deficiency and other factors. Overall the pharmacodynamic effects of the SQ rituximab formulation compared well with the IV formulation, with eight of the 10 patients with full data sets available for analysis after 1 month experiencing a significant increase in ADAMTS13 activity after one or more SQ rituximab treatments, while all 10 patients had undetectable CD19 (+) B lymphocytes; all 10 patients had ADAMTS13 activity >25 IU/dL at 3 months and persistently undetectable CD19 (+) B cells on flow cytometry at that time. All 12 patients did exhibit normal ADAMTS13 activity during the study, including the two patients without full data sets available for analysis. One patient had an injection site reaction that was treated with corticosteroids, and otherwise no safety events were reported, including no infections. At the time of publication, this is the only study specifically investigating the utilization of subcutaneous rituximab for the treatment of iTTP.

The question of whether patients should be administered maintenance rituximab regardless of the presence of clinical or ADAMTS13 relapse of iTTP remains somewhat controversial. Several studies have reported good outcomes for patients treated with maintenance rituximab, commonly in intervals of six or fewer months given the pharmacokinetics of the drug as described in the section above regarding clinical trials in iTTP (Ojeda-Uribe et al., 2010; Bhagirath et al., 2012; Saleem et al., 2019). However, the decision to pursue rituximab maintenance is nuanced. The logistics of weekly transfusions, the choice of dosing regimen if maintenance is pursued, the relative paucity of evidence demonstrating a clear benefit of maintenance rituximab treatment, adverse effects from rituximab treatment itself, and variability in practitioners’ utilization of tools such as routine ADAMTS13 activity testing during periods of iTTP remission are all contributing factors. In practice, a reasonable approach is to monitor iTTP patients’ ADAMTS13 activity level, complete blood counts, comprehensive metabolic panel, lactate dehydrogenase, reticulocyte count, and haptoglobin every 3 months while they are in remission, and provide repeated CD20 directed treatment when ADAMTS13 levels drop as confirmed by repeat testing in a shorter time frame, regardless of the presence of clinical relapse.

#### 4.1.4 Refractoriness and resistance to rituximab therapy for iTTP

Though generally well tolerated with a fairly robust line of evidence demonstrating its general efficacy in the treatment of iTTP, rituximab, like any drug, is not universally beneficial to all patients. Adverse effects of the drug–ranging from injection site reactions to anaphylaxis–are not uncommon and can be potentially treatment limiting (Anderson et al., 1997; Khellaf et al., 2014; Mahevas et al., 2013; Xue and Qian, 2025; Weisinger et al., 2025a). Also, a proportion of iTTP patients, unfortunately, do not derive the remission benefit from rituximab that others experience. For such patients, alternative immunosuppressive approaches have been utilized with agents such as bortezomib (Giannotta et al., 2025; Wali et al., 2023; Lee et al., 2024; Yin et al., 2022; Maschan et al., 2021), vincristine (da Rocha Ribas et al., 2024), daratumumab (Aggarwal et al., 2023), N-acetylcysteine (Beyler and Demir, 2023), and mycophenolate mofetil (Ahmad et al., 2007). To date, the factors that contribute to refractoriness and resistance to rituximab are not fully known. As mentioned in a previous section, factors such as age, blood type, and previous episodes of iTTP appear to be associated with the risk of recurrence. In this section, we review two important studies from the United States that appear to shed some light on the patient populations at risk for suboptimal responses to rituximab as stratified by race.

In 2022, the United States Thrombotic Microangiopathies Consortium conducted a retrospective analysis on 645 patients who experienced a total of 1,049 episodes of iTTP from1995 to 2020, stratifying patients by race and whether or not they were treated with rituximab (Chaturvedi et al., 2022). Of the entire cohort, 44% of Black patients were treated with rituximab, in contrast to 37% of White patients. All patients who did receive rituximab were treated with four doses of 375 mg/m2 IV; 96.2% of patients received four doses weekly and the remainder received shorter intervals between doses. For patients with *de novo* disease, rituximab improved relapse-free survival, but did not reach the level of statistical significance, regardless of race. However, when all episodes of iTTP were considered, White patients who received rituximab had a statistically significant improvement in relapse-free survival, but Black patients did not. The study was confounded by its retrospective nature, as it was not possible to control for variables such as disease severity at presentation as a decision-influencing factor regarding the use of rituximab.

In 2025, the United States Thrombotic Microangiopathy Alliance (USTMA) published updated findings on relapse-free survival amongst iTTP patients treated with rituximab and stratified by race (Fatola et al., 2025). A retrospective analysis of 310 patients in the USTMA registry who received rituximab between 2004 and 2019 was pursued as well as a prospective study with a cohort of 97 patients who presented to Johns Hopkins University and the University of Minnesota between 2004 and 2023. All patients in the cohort received 375 mg/m^2^ IV rituximab weekly for 4 weeks. In the retrospective cohort, the median clinical relapse-free survival was 6.0 years after the first rituximab treatment, but 2.1 years after the second or subsequent rituximab-treated episode of clinical iTTP relapse. Interestingly, Black patients had a significantly higher risk of clinical relapse after the second or subsequent rituximab treatment courses, and Black race in addition to previous relapsed iTTP and a second or subsequent course of rituximab were found to be statistically significant predictors of clinical relapse. The prospective analysis corroborated the race-associated data, as overall relapse-free survival was not significantly different after the first and subsequent rituximab treatment for White patients, while Black patients experienced progressively and statistically significantly shorter periods of overall relapse-free survival with each rituximab treatment course.

### 4.2 Obinutuzumab and ofatumumab

The role of alternative CD20-targeting antibodies has largely been to replace rituximab for patients refractory or resistant to rituximab utilized for iTTP treatment. We will outline the available data regarding the efficacy of these agents separately in this section. As discussed above, refractoriness and resistance to rituximab treatment does occur in the management of patients with iTTP, and for these patients, Obinutuzumab and ofatumumab can be utilized as alternative anti-CD20 antibody options. Obinutuzumab is a fully humanized type-II monoclonal anti-CD20 antibody, designed for relapsing and refractory diseases after treating with rituximab-based regimens (Davies et al., 2022). Obinutuzumab has glycoengineered non-fucosylated sugars on the Fc portion, leading to enhanced affinity for FcγRIIIa receptors on immune effector cells, and subsequently more potent antibody dependent cellular cytotoxicity (ADCC) and antibody-dependent phagocytosis (ADP). Obinutuzumab is superior to rituximab in B cell depletion (Mossner et al., 2010) and activity and has demonstrated promising outcomes in indolent B-cell malignancies (Davies et al., 2022), composing the first line therapy in chronic lymphocytic leukemia (CLL) (Shadman, 2023) and part of an alternative anti-CD20 reagent combined with chemotherapy in aggressive follicular lymphoma (FL) (Jacobsen, 2022; Chu et al., 2024; Kahl, 2021). Ofatumumab is a fully human type I anti-CD20 antibody that is also used as first-line therapy for CLL patients, particularly older patients and those with comorbid conditions (Abrisqueta et al., 2024; Chavez et al., 2024; Traynor, 2009; Reagan and Castillo, 2011). Both Obinutuzumab and ofatumumab have also been utilized for the treatment of non-neoplastic autoimmune diseases such as multiple sclerosis (Freedman et al., 2025; Yang et al., 2025; Azargui et al., 2025; Robak, 2008), autoimmune hemolytic anemia (Li et al., 2024; Herishanu et al., 2021; Poulet et al., 2019; Nader et al., 2013), and ITP (Yokota et al., 2025; Blase et al., 2022).

Obinutuzumab in the treatment of iTTP patients was first reported in a few case reports and patient series. In a multicenter cohort of UK iTTP patients (Doyle et al., 2022; Maitta, 2022), the usage of 26 episodes of Obinutuzumab (8/26) and ofatumumab (16/26) treatment in 15 iTTP patients was reported. Complete remission was achieved in 24/26 episodes and partial remission in 2/26 episodes. The median time to peak ADAMTS13 activity response was 120 days. Among the eight patients who received Obinutuzumab, one experienced ADAMTS13 relapse, but the median follow up of non-relapse patients was 7.7 months compared to the one relapse who experienced a relapse-free survival (RFS) of 15.4 months. In this series, some data of patients administered the two alternative anti-CD20 monoclonal antibodies were analyzed together. Weisinger et al. reported another retrospective analysis including 60 iTTP patients receiving Obinutuzumab treatment due to rituximab intolerance (25/60), refractoriness to rituximab (32/60), or both (3/60), recruited from the CNR-MAT registry in France (Weisinger et al., 2025b). With a median follow up of 11 months after Obinutuzumab initiation, the response rate was 85% (51/60); 72% had complete response with normalized ADAMTS13 activity ≥50%. The median times to reach a partial response (ADAMTS13 activity ≥20%) and complete response (ADAMTS13 activity ≥50%) were 36 days and 76 days, respectively. In this cohort, it was also observed that the rate of efficacy in ADAMTS13 activity improvement among the patients formerly unresponsive to rituximab was significantly lower than among the patients intolerant to rituximab, and most of the patients refractory to Obinutuzumab (8/9, 89%) were also unresponsive to rituximab. Three patients experienced ADAMTS13 (2/3) and clinical (1/3) relapses and needed further management. The efficacy and safety of Obinutuzumab was also reflected in some individual iTTP cases, who resorted to Obinutuzumab for the reason of serum sickness disease, allergy, or rituximab resistance with detectable anti-rituximab antibody (Schimpf et al., 2025; Robertz et al., 2019; Ahmadpoor et al., 2021), but the results of long-term follow up still need to be elucidated. It is noted that, in the cohort of the CNR-MAT registry, B cell depletion results were available in 34 patients, all showing peripheral CD19^+^ B-cell lymphocytes <1% within 2 months after Obinutuzumab initiation (Weisinger et al., 2025b). In a case report of a patient who developed rituximab resistance due to anti-rituximab antibody formation, peripheral CD19^+^ B-cell lymphocytes remained undetectable 4 months after infusion of Obinutuzumab (Ahmadpoor et al., 2021).

In the above reports, Obinutuzumab was given intravenously at an interval of 1–2 weeks, at a dose of 1,000 mg IV, just as given in regimens for B-cell malignancies. It is usually administered over 2 days, 100 mg on day 1 and 900 mg on day 2 (Doyle et al., 2022; Weisinger et al., 2025b; Schimpf et al., 2025; Robertz et al., 2019; Ahmadpoor et al., 2021). The patients received a median of 3 (IQR, 1–4) doses of Obinutuzumab in the cohort of the CNR-MAT registry (Weisinger et al., 2025b), and 2–3 infusions in the patients from the UK TTP registry and all case reports. In the first UK trial (Doyle et al., 2022), there were four adverse effects reported: two infections and two mild infusion-related reactions; because the data combined results of both Obinutuzumab and ofatumumab, specific attribution to either drug could not be assigned. In the French cohort, side effects were recorded in 11 patients, of which 6 were Grade 1–2 infusion-related reactions, as well as mild symptoms of headache, chest discomfort, hypertension and fatigue (Weisinger et al., 2025b). No adverse reactions to Obinutuzumab were reported within the four patients from the 3 case reports (Schimpf et al., 2025; Robertz et al., 2019; Ahmadpoor et al., 2021).

The French CNR-MAT reported in 2020 on the efficacy of an intensive regimen of rituximab (multiple infusions of 375 mg/m^2^ IV more often and for a longer duration than 4 weeks) for patients who did not achieve improved ADAMTS13 activity levels following standard rituximab treatment (Barba et al., 2021). Notably in this cohort of 13 patients, one patient was found to essentially be refractory to the intensive regimen and was subsequently treated with “four ofatumumab infusions weekly (300 mg–1000 mg/week x3)”. ADAMTS13 activity recovery was achieved. Ofatumumab has also reportedly been successfully utilized in a patient with multiple relapses of iTTP (Al-Samkari et al., 2018) and another patient who experienced anaphylaxis associated with rituximab (Crowley et al., 2018). In the latter case report, ofatumumab was given intravenously at doses of 300 mg on day 1, 1,000 mg on day 8, and 1,000 mg on day 15, notably different from regimens given in the context of other diseases (Hillmen et al., 2015; Robak et al., 2017; Farez et al., 2019; Gavriatopoulou et al., 2017).

Obinutuzumab and ofatumumab appear to be effective alternative anti-CD20 therapies for iTTP patients intolerant and refractory to rituximab. Whether these alternative CD20 targeting therapies are effective during the acute phase is remained to be answered, and the long-term efficacy and safety of their use in the treatment of iTTP patients still needs to be systemically clarified with multicenter clinical trials (Weisinger et al., 2025a). However, given their generally benign safety profile and utility in non-iTTP patients as well as their mechanistic similarities to rituximab, it is also reasonable to consider utilization of Obinutuzumab and ofatumumab in the up-front setting for patients who are not candidates for rituximab therapy but for whom CD20-directed therapy is not contraindicated.

## 5 Biosimilars

With an increasing number of biologics approved and patents expiring, biosimilars have become increasingly common. Biosimilars are biological products that are developed with no clinically meaningful difference in efficacy, safety, purity, and pharmacodynamics as the originator product. Although not an exact copy, a biosimilar is “highly similar” to the originator product (Declerck et al., 2017). Biosimilars differ from generic products, which are typically replications of small molecules, because biologics are large, complex products produced from a living cell leading to natural variance in protein folding and glycosylation (Mascarenhas-Melo et al., 2024). FDA approval for biosimilar products is different than a new molecule or generic product; the biosimilar manufacturer only must show proof of biosimilarity, not independently establish efficacy, often leading to a quicker, abbreviated approval process. The backbone of biosimilar approvals are analytic studies, which evaluates the structural and functional similarities between the biosimilar and originator product. Additional studies may include animal studies and clinical pharmacologic studies–to prove that the biosimilar moves through the body in the same way as the originator product (Shelbaya et al., 2021). There is a similar process for the EMA where the manufacturer of the biosimilar product must complete clinical and non-clinical comparative studies to ensure similar efficacy and quality to the originator product (Vital et al., 2013).

Rituximab-abbs, rituximab-pvvr, and rituximab-arrx are rituximab biosimilars currently available in the US. Slight differences exist between the biosimilars and originator product, but the overall amnio acid structure is the same between products. The largest difference between products is variable glycosylation. Rituximab-pvvr has the highest afucosylated glycan and sialylation levels compared to other biosimilars which may be associated with higher antibody-dependent cellular cytotoxicity, while higher mannose glycan levels and terminal galactosylation is seen with rituximab-abbs and rituximab-arrx, respectively (Na et al., 2025). These differences do not translate into differences in efficacy. When evaluated in treatment-naïve follicular lymphoma (FL), rituximab-pvvr showed non-significant differences in overall response rate compared to rituximab; there was no significant difference in adverse events between the biosimilar and originator product (Sharman et al., 2020). Rituximab-abbs has had similar results when evaluated in diffuse large B-cell lymphoma and follicular lymphoma; in both disease states the biosimilar was found to be non-inferior in terms of safety and efficacy to the originator product (Wong et al., 2024; Kwak et al., 2022). The JASMINE trial found rituximab-arrx to be non-inferior in both efficacy and safety in asymptomatic FL (Niederwieser et al., 2020). Minimal trials have been conducted outside of oncologic indications, but when evaluated in rheumatoid arthritis, the biosimilars yielded similar pharmacokinetic, pharmacodynamic, safety, and efficacy results as the originator product (49,52). Relevant to this review, in a single-center study, rituximab was compared to rituximab-abbs in iTTP (Stubbs et al., 2019). Between the two groups, there was no statistical significance between platelet counts at day 1, day 28, or 3 months post-infusion. Similarly, ADAMTS13 activity was similar across time points between rituximab and rituximab-abbs. The use of rituximab-abbs did exhibit a significant cost savings compared to use of rituximab which may allow for increased access to urgent therapy. Despite limited data for use, rituximab biosimilars appear to have similar safety and efficacy in iTTP.

Initial comparator studies indicated similar safety profiles between biosimilars and the originator product, but real-world data may support slight differences in safety profiles. Post-marketing reporting through FAERS database indicates there may be a trend to higher rates of pruritus and infusion-related reactions with rituximab-pvvr compared to the originator product (Xue and Qian, 2025). A single-center retrospective study reported an increase of infusion related reactions after transitioning from the originator product to rituximab-pvvr, though this was not statistically significant (Triesel et al., 2022). Additional larger, prospective studies are necessary to detect any significant difference in safety with rituximab biosimilars.

## 6 Discussion

In this review, we discuss the role of CD20-directed therapy in the treatment of iTTP in the modern era. Utilization of rituximab has become standard of care in the up-front setting at most major academic centers around the world. Knowledge of the available data to date regarding the utility of these agents and the potential risks of their use guides clinical decisions for providers caring for iTTP patients and informs future directions of research to optimize their efficacy and expand the patient populations for which anti-CD20 antibodies help induce lasting remissions from the disease. It is our hope that this relatively comprehensive review on the state-of-the-art regarding CD20-directed therapy will help to serve this role in the literature.

Though there have been dozens of studies published to date regarding the use of anti-CD20 antibodies in the treatment of iTTP, particularly rituximab, a great deal of heterogeneity exists in the literature, as we have endeavored to make clear above. The majority of studies to date have utilized the same treatment regimens used in hematologic malignancies for iTTP. It is not entirely clear if this approach is redundant and confers additional risk of adverse effects from CD20-targeted therapy to patients that may be mitigated by the use of lower doses. In our own practice, we do use a lower dose of rituximab than used for lymphoma similar to those utilized for patients with other autoimmune diseases. Recently, we have been favoring utilization of 1,000 mg IV rituximab on days 1 and 15, particularly in the outpatient setting, and have found it to be efficacious with no significant adverse events in our experience. We also routinely monitor ADAMTS13 activity levels for iTTP patients in remission to determine if and when to pursue pre-emptive treatment with rituximab to prevent clinical iTTP relapse. As should be clear, however, additional data from larger prospective studies with fewer clinical variables between patient populations would provide further support for such “alternative” dosing regimens and to inform standard approaches to the long-term management of iTTP patients. Similarly, the definition of refractoriness to CD20-directed therapy remains ambiguous, and future studies could help clinicians to determine when to consider alternative anti-CD20 agents and/or different classes of immunosuppressive agents.

As our aim was to present the data from available trials and patient populations, in part to demonstrate the heterogeneity of the variables in these reports, we did not include meta-analyses regarding the efficacy of rituximab and the other anti-CD20 antibodies in iTTP. Work by others, however, largely corroborates our conclusions that CD20-directed therapy is relatively safe and significantly improves relapse-free survival in iTTP patients, and when viewed in aggregate, significantly improves mortality as well (Owattanapanich et al., 2019). The reader is encouraged to peruse the literature cited in this review in deciding their approach to treating iTTP and identifying future needs in this space. Nonetheless, we conclude that CD20-directed therapy plays an important role in the management of iTTP patients and should continue to be utilized as better and more targeted therapies continue to evolve.
